# Increased METTL3-mediated m^6^A methylation inhibits embryo implantation by repressing HOXA10 expression in recurrent implantation failure

**DOI:** 10.1186/s12958-021-00872-4

**Published:** 2021-12-14

**Authors:** Pingping Xue, Wenbo Zhou, Wenqiang Fan, Jianya Jiang, Chengcai Kong, Wei Zhou, Jianmei Zhou, Xiaoyang Huang, Haiyan Yang, Qian Han, Bin Zhang, Lingyun Xu, Bin Yu, Li Chen

**Affiliations:** 1 Department of Reproductive Medicine Center, Changzhou Maternity and Child Health Care Hospital Affiliated to Nanjing Medical University, Changzhou, 213000 China; 2 Department of Medical Genetics, Changzhou Maternity and Child Health Care Hospital Affiliated to Nanjing Medical University, Changzhou, 213000 China; 3 Department of Mammary Surgery, Changzhou No.2 People’s Hospital Affiliated to Nanjing Medical University, Changzhou, 213000 China

**Keywords:** METTL3, m^6^A methylation, HOXA10, embryo implantation, recurrent implantation failure

## Abstract

**Background:**

Recurrent implantation failure (RIF) is a major limitation of assisted reproductive technology, which is associated with impaired endometrial receptivity. Although N^6^-methyladenosine (m^6^A) has been demonstrated to be involved in various biological processes, its potential role in the endometrium of women with RIF has been poorly studied.

**Methods:**

Global m^6^A levels and major m^6^A methyltransferases/demethylases mRNA levels in mid-secretory endometrium from normal and RIF women were examined by colorimetric m^6^A quantification strategy and quantitative real-time PCR, respectively. The effects of METTL3-mediated m^6^A modification on embryo attachment were evaluated by an vitro model of a confluent monolayer of Ishikawa cells co-cultured with BeWo spheroids, and the expression levels of homeo box A10 (HOXA10, a well-characterized marker of endometrial receptivity) and its downstream targets were evaluated by quantitative real-time PCR and Western blotting in METTL3-overexpressing Ishikawa cells. The molecular mechanism for METTL3 regulating HOXA10 expression was determined by methylated RNA immunoprecipitation assay and transcription inhibition assay.

**Results:**

Global m^6^A methylation and METTL3 expression were significantly increased in the endometrial tissues from women with RIF compared with the controls. Overexpression of METTL3 in Ishikawa cells significantly decreased the ration of BeWo spheroid attachment, and inhibited HOXA10 expression with downstream decreased β3-integrin and increased empty spiracles homeobox 2 expression. METTL3 catalyzed the m^6^A methylation of HOXA10 mRNA and contributed to its decay with shortened half-life. Enforced expression of HOXA10 in Ishikawa cells effectively rescued the impairment of METTL3 on the embryo attachment ***in vitro***.

**Conclusion:**

Increased METTL3-mediated m^6^A modification represents an adverse impact on embryo implantation by inhibiting HOXA10 expression, contributing to the pathogenesis of RIF.

## Background

Embryo implantation is an important and complex process in the establishment of pregnancy in mammals, which requires the simultaneous development of high-quality embryos and endometrial receptivity [1–3]. Endometrium is one of the most dynamic tissues in human body. Under the action of steroids during sexual cycle, endometrium undergoes cyclic developmental changes and highly ordered differentiation, leading to be receptive to blastocyst implantation ∼6 days after ovulation and remains receptive for 4 days (cycle days 20-24) [4, 5]. Endometrial receptivity deficiency may be the critical factor for women with recurrent implantation failure (RIF) who have high-quality embryos but undergo repeated implantation failure following *in vitro* fertilization-embryo transplantation (IVF-ET) treatment [6]. Several different signaling pathways and their associated genes have been demonstrated to be involved in the adjustment of endometrial receptivity [5, 7], in which homeo box A10 (HOXA10) has emerged as an important and well-characterized biomarker. The expression of HOXA10 is dynamic through the menstrual cycle and significantly increased at the time of implantation with increased progesterone levels [8, 9]. The gene *HOXA10* has a highly conserved homeodomain that specifically recognizes the TTAT sequence in the promoter of downstream target genes, leading to expression changes of target genes, including β3-integrin (ITGB3) and empty spiracles homeobox 2 (EMX2) [10–12]. Studies have shown that ITGB3 expression is directly up-regulated by HOXA10 [10], whereas EMX2 expression is inhibited by HOXA10 [12]. ITGB3 is a transmembrane glycoprotein that presents on the surface of cells, which participates in cell adhesion and cell-surface-mediated signaling during embryo implantation [13–15]. EMX2 is a crucial transcription factor necessary for reproductive tract differentiation and development, but changes in endometrial EMX2 expression levels usually lead to abnormalities of the endometrium [16, 17]. Accumulating studies indicate that decreased HOXA10 expression contributes to the failure of embryo implantation [18–21]. However, the expression of HOXA10 and its underlying mechanisms for epi-transcriptomic regulation of HOXA10 in RIF remain to be characterized.

N^6^-methyladenosine (m^6^A) is the most abundant internal modification in messenger RNAs (mRNAs). The m^6^A modification is catalyzed by “writers” methyltransferases, including methyltransferase-like 3 (METTL3), methyltransferase-like 14 (METTL14), RNA binding motif protein 15 (RBM15) and Wilms tumor 1-associated protein (WTAP). Meanwhile, m^6^A modification can be removed by “erasers” demethylase, such as fat mass and obesity-associated protein (FTO) and AlkB homolog 5 (ALKBH5). In addition, m^6^A “readers” are responsible for recognition of the m^6^A modification [22]. Members of every classes of m^6^A regulators cooperatively participate in the regulation of mRNA stability and translation, affect gene expression output and thus play an important role in physiological and pathological conditions [22–26]. In the m^6^A methyltransferase complex, METTL3 functions as the the catalytic core while METTL14 serves as the RNA-binding platform [27]. METTL3 is a transferase that methylates mRNA, identifies methylated mRNA, and regulates mRNA translation. Recently, accumulating studies have identified multiple roles and molecular mechanisms associated with METTL3 in various biological processes [28–30]. However, whether METTL3-mediated m^6^A modification is involved in the regulation of endometrial receptivity and how this relates to RIF remains unclear.

Herein, we found that both the levels of global mRNA m^6^A methylation and METTL3 were significantly elevated in the endometrial tissues from RIF patients compared with the controls. Overexpression of METTL3 in Ishikawa cells significantly decreased the ration of BeWo spheroid attachment. METTL3 catalyzed the m^6^A methylation of HOXA10 mRNA and contributed to its decay with shortened half-life. Enforced expression of HOXA10 in Ishikawa cells effectively rescued the impairment of METTL3 on the BeWo spheroid attachment *in vitro*. Our study reveals that METTL3-mediated m^6^A modification could have an impact on embryo implantation and may contribute to the pathogenesis of RIF.

## Materials and Methods

### Patient samples and ethical approval

The patients enrolled in this study were recruited from *in vitro* fertilization unit of Reproductive Medicine Center of the Affiliated Changzhou Maternity and Child Health Care Hospital of Nanjing Medical University. All the endometrial samples were collected with written informed consent of the patients, and approval from the Scientific Research Ethics Committee was obtained for this study (2020103). The mid-secretory phase endometrial tissues were collected by endometrial biopsy from normal women and women with RIF according to the criteria described previously [19]. The normal control group was composed of women who were infertile due to male factors and proved to be fertile after the IVF-ET treatment. RIF was defined as the absence of implantation following two fresh or frozen embryo replacement cycles, during which at least four embryos with good quality were transferred to uterus. Women with endometriosis, adenomyosis, hydrosalpinx, uterine malformation, endometrial polyps or autoimmune disease were not included. The details of these patients are summarized in Table 1.

**Table 1:** Demographic details of the participants in this study.

Analyzed items	Normal groups (n=13)	RIF patients (n=13)	*P*-valve
Age (years)	29.38 ± 3.89	31.54 ± 3.27	0.1554
Body mass index (kg/m^2^)	22.90 ± 2.31	21.85 ± 2.15	0.2626
Menstrual cycle (days)	32.15 ± 8.63	29.92 ± 4.16	0.4277
Endometrial thickness (mm)	11.36 ± 2.10	9.96 ± 1.39	0.0663
FSH (mIU/mL, Day 3)	7.74 ± 1.60	8.08 ± 1.97	0.6454
LH (mIU/mL, Day 3)	5.27 ± 1.89	5.95 ± 1.88	0.3866
Estrogen (pg/mL, Day 3)	40.02 ± 17.09	32.69 ± 11.76	0.2328
AFC (R, Day 3)	7.50 ± 2.87	5.50 ± 0.50	0.4058
AFC (L, Day 3)	7.50 ± 2.74	6.67 ± 0.94	0.6520
Number of total transferred embryos	1.69 ± 0.72	6.23 ± 2.01	3.16E-08
Number of per transferred embryos	1.46 ± 0.46	1.73 ± 0.24	0.0854

### Cell culture

The human endometrial adenocarcinoma cell line Ishikawa was purchased from the Cell Bank of Type Culture Collection of the Chinese Academy of Sciences (Shanghai, China) and maintained in MEM medium (Thermo Fisher Scientific, Waltham, MA, USA). The human placental choriocarcinoma cell line BeWo was purchased from the American Type Culture Collection (Manassas, VA, USA) and maintained in Ham's F-12K (Kaighn's) medium (Thermo Fisher Scientific). These mediums contain 10% fetal bovine serum (FBS; Thermo Fisher Scientific), 100 U/mL penicillin, and 100 mg/mL streptomycin (HyClone, South Logan, UT, USA). Cells were incubated at 37°C with 5% CO_2_. Actinomycin D (S8946; Selleck Chemicals) was added for the indicated times at a final concentration of 10 μg/mL for the transcription inhibition assay.

### Quantification of m^6^A RNA methylation

Total RNA was extracted using TRIzol reagent (Invitrogen, Carlsbad, CA, USA), followed by the purification of polyadenylated mRNA using Dynabeads mRNA Purification Kit (Thermo Fisher Scientific) according to manufacturer’s protocol. An m^6^A RNA Methylation Assay Kit (Abcam, Cambridge, MA, USA) was used to evaluate the m^6^A content of total RNA according to the manufacturer’s instructions, as previously reported [31]. Equal amounts of total RNA (200 ng) were bound to strip wells using a RNA high binding solution. The m^6^A was captured and detected using the specific capture antibody and detection antibody. Then, the detected m^6^A signal was enhanced using enhancer solution, and quantified colorimetrically after adding color developing solutions by reading the absorbance at a wavelength of 450 nm in a microplate spectrophotometer.

### Dot blotting assay

A dot blotting assay was performed essentially as previously reported [32]. Total RNA or poly (A) + mRNA was isolated as described above. Equal amounts of total poly (A) + mRNA samples (2 μg) were denatured at 65°C for 5 min. Then the samples were loaded onto nylon membranes (GE Healthcare, USA) with ice-cold 20× saline sodium citrate solution (Sigma Aldrich) in a dot blot apparatus (Bio-Rad, USA). The membranes were then UV-crosslinked for 5 min, blocked with 5% non-fat milk for 1 hour, incubated with an m^6^A antibody (1:400; ab151230, Abcam) overnight at 4 °C and horseradish peroxidase-conjugated anti-rabbit IgG for 1 hour at room temperature, and finally detected with a 3,3’-diaminobenzidine peroxidase substrate kit. At the same time, the same poly (A) + mRNA (2 μg) samples were spotted onto membranes, UV-crosslinked twice, stained with 0.02% methylene blue in 0.3 M sodium acetate for 2 hours, and washed with ribonuclease-free water for 5 hours, followed by the scanning to indicate the total content of input RNA.

### Quantitative real-time PCR (qRT-PCR)

Total RNA was lysed using TRIzol reagent and used for the synthesis of cDNA with a One-Step RT-PCR Kit (Thermo Fisher Scientific). Reactions of qRT-PCR were performed using the ABI Vii7 system (Applied Biosystems, USA). GAPDH was used as a housekeeping gene. Relative gene expression was calculated by the 2^-△△CT^ cycle threshold method [33]. The primers used for qRT-PCR analysis are listed in Table 2.

**Table 2:** Oligonucleotide primer sequences for qRT-PCR.

Gene	Forward primer (5’-3’)	Reverse primer (5’-3’)
METTL3	CAAGCTGCACTTCAGACGAA	GCTTGGCGTGTGGTCTTT
METTL14	CTGGGGAGGGGTTGGACCTT	CCCCGTCTGTGCTACGCTTC
RBM15	TCCCACCTTGTGAGTTCTCC	GTCAGCGCCAAGTTTTCTCT
WTAP	CTTCCCAAGAAGGTTCGATTGA	TCAGACTCTCTTAGGCCAGTTAC
VIRMA	AATCCTGTGGGAAGATCAGC	ACACGTAAGGCAGTGGTAAG
FTO	CCAGAACCTGAGGAGAGAATGG	CGATGTCTGTGAGGTCAAACGG
ALKBH5	CCAGCTATGCTTCAGATCGCCT	GGTTCTCTTCCTTGTCCATCTCC
HOXA10	AGATTAGCCGCAGCGTCCAC	GTAACGGCCCAGGAGATGGC
ITGB3	TGTGTCCGCTACAAGGGGGA	TGTAGGGCTCCCCGGTCAAA
EMX2	CGGTAGGGGCGTCTACTCCA	TCGGATCCGCTTGGGCTTTC
GAPDH	TGACTTCAACAGCGACACCCA	CACCCTGTTGCTGTAGCCAAA

### Cell transfection and stable cell line construction

Recombinant lentiviruses expressing wild-type METTL3 (OE-METTL3-WT) or control (OE-con), catalytic domain mutant METTL3 (D395A and W398A; OE-METTL3-Mut) and HOXA10 (OE-HOXA10) were purchased from Biosmedi (Shanghai, China). The cell line Ishikawa was transfected with concentrated lentiviruses (OE-METTL3-WT, 50 MOI; OE-METTL3-Mut, 50 MOI; OE-HOXA10 50 MOI), and stable cell lines were selected by treatment with puromycin for 2 weeks.

### Western blotting analysis

Western blotting analysis was performed as previously described [34] using antibodies against METTL3 (1:1,500; ab221795, Abcam), HOXA10 (1:2,000; A8550, Abclonal), ITGB3 (1:1,000; ab119992, Abcam), EMX2 (1:1,500; ab171818, Abcam) and GAPDH (1:6,000; KC-5G5, Aksomics). GAPDH was used as an endogenous control to normalize protein loading. The relative band intensities were measured using a quantitative scanning densitometer and image analysis software, ImageJ.

### *In vitro* embryo implantation assay

We used multicellular spheroids of human placental choriocarcinoma BeWo cells co-cultured with a confluent monolayer of endometrial adenocarcinoma Ishikawa cells as an *in vitro* model of embryo attachment [19]. First, a single-cell suspension of BeWo cells was placed in a 35 mm^2^ culture dish pre-coated with an anti-adhesive polymer poly-2-hydroxyethyl methacrylate (Sigma Aldrich). Multicellular spheroids of BeWo cells were induced after 48 hours of culture and 150-200 μm in diameter. Meanwhile, E2 (10^-8^ M) and P4 (10^-6 ^M) were added into the medium of the monolayer stable METTL3- and/or HOXA10-overexpressing Ishikawa cells after reaching 70%–80% confluence in a 24-well culture plate. Simultaneously, BeWo spheroids were transferred onto the confluent monolayer of Ishikawa cells. After incubation at 37°C for 2 hours, cells were washed with phosphate buffer saline containing 0.1 mg /L Ca^2+^ and Mg^2+^ to remove the unattached spheroids. The attached spheroids were then counted under a light microscope, and the adhesion rate was expressed as a percentage of the total number of BeWo spheroids added onto the Ishikawa monolayer (% adhesion).

### Methylated RNA immunoprecipitation (Me-RIP) assay

A previously described procedure was used for Me-RIP [32]. The Dynabeads mRNA Purification Kit (Thermo Fisher Scientific) was used to purify mRNA from total RNA and the RNA quality was analyzed by NanoDrop 2000. Then, a Magna MeRIP™ m^6^A Kit (17-10499, Merck Millipore) was used to measure the changes in the m^6^A levels of the mRNA according to the manufacturer’s protocol. We saved 0.5 μg of the mRNA as input and used the remaining mRNA for m^6^A immunoprecipitation. After being immunoprecipitated with Magna ChIP protein A/G Magnetic Beads and eluted twice with elution buffer, the m^6^A-precipitated RNA was recovered by ethanol precipitation. The RNA concentration was measured with NanoDrop 2000 and the immunoprecipitated m^6^A RNA was used as templates for qRT-PCR.

## Statistical analysis

Data are presented as mean ± SD of at least three independent experiments. Statistical analyses were performed using GraphPad Prism 9 software (La Jolla, CA, USA). Differences between group means were evaluated with the Student’s *t-*test or one-way analysis of variance. *P* < 0.05 shows a statistical significance.

## Results

### Upregulation of m^6^A modification and METTL3 in the endometrial tissues of women with RIF

**Fig. 1:**
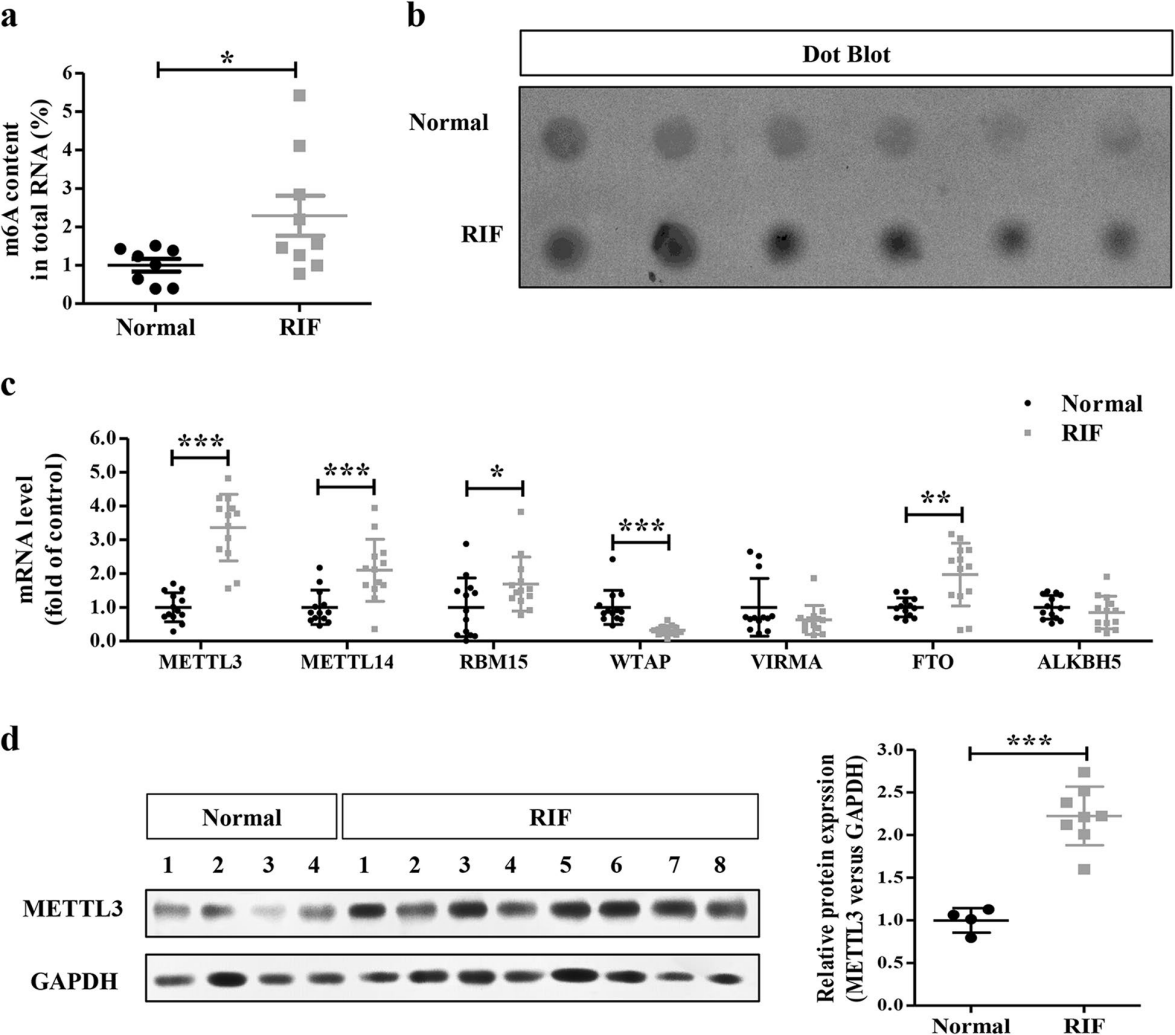
**Increased m**^**6**^**A RNA methylation and METTL3 expression in RIF endometrial tissues. a** The levels of m^6^A RNA methylation in the endometrial tissues from RIF patients (n=9) and normal control women (n=8) were evaluated by the m^6^A RNA Methylation Assay Kit. **b** The m^6^A levels in the endometrial tissues from RIF patients (n=6) and health control women (n=6) were evaluated by dot blotting assay. **c** The mRNA levels of major m^6^A methyltransferases (METTL3, METTL14, RBM15, WTAP and VIRMA) and demethylases (FTO and ALKBH5) in the endometrial tissues from RIF patients (n=13) and normal control women (n=13) were detected by qRT-PCR. **d** The protein levels of METTL3 in the endometrial tissues from RIF patients (n=8) and normal control women (n=4) were detected by Western blotting. *P<0.05, **P<0.01, ***P<0.001compared with the controls

To explore the potential role of m^6^A modification in RIF, we first examined global m^6^A levels in total RNA from mid-secretory phase endometrial tissues of normal and RIF women by colorimetric m^6^A quantification strategy. We found that endometrial m^6^A levels were significantly increased in the RIF patients than in the controls (Fig. 1a). This increase was further confirmed by dot blotting assay (Fig. 1b).

Then, we detected the mRNA levels of major m^6^A methyltransferases (METTL3, METTL14, RBM15, WTAP and VIRMA) and demethylases (FTO and ALKBH5) in the normal and RIF endometrial tissues. The m^6^A methyltransferases (METTL3, METTL14, and RBM15) and demethylase FTO were significantly increased in the RIF endometrial tissues compared with normal controls, while the m^6^A methyltransferase WTAP was significantly decreased in the RIF endometrial tissues (Fig. 1c). However, there were no significant differences in the mRNA levels of VIRMA or ALKBH5 between normal and RIF patients.

Considering the increased m^6^A modification in RIF endometrium and the catalytic abilities of these m^6^A regulators, we selected METTL3 as the candidate molecule for further studies of aberrant m^6^A modification in RIF. Protein levels of METTL3 in the RIF endometrial tissues were significantly increased in comparison to normal controls (Fig. 1d), similar to the result obtained from qRT-PCR.

### METTL3 overexpression impairs embryo attachment *in vitro*

**Fig. 2:**
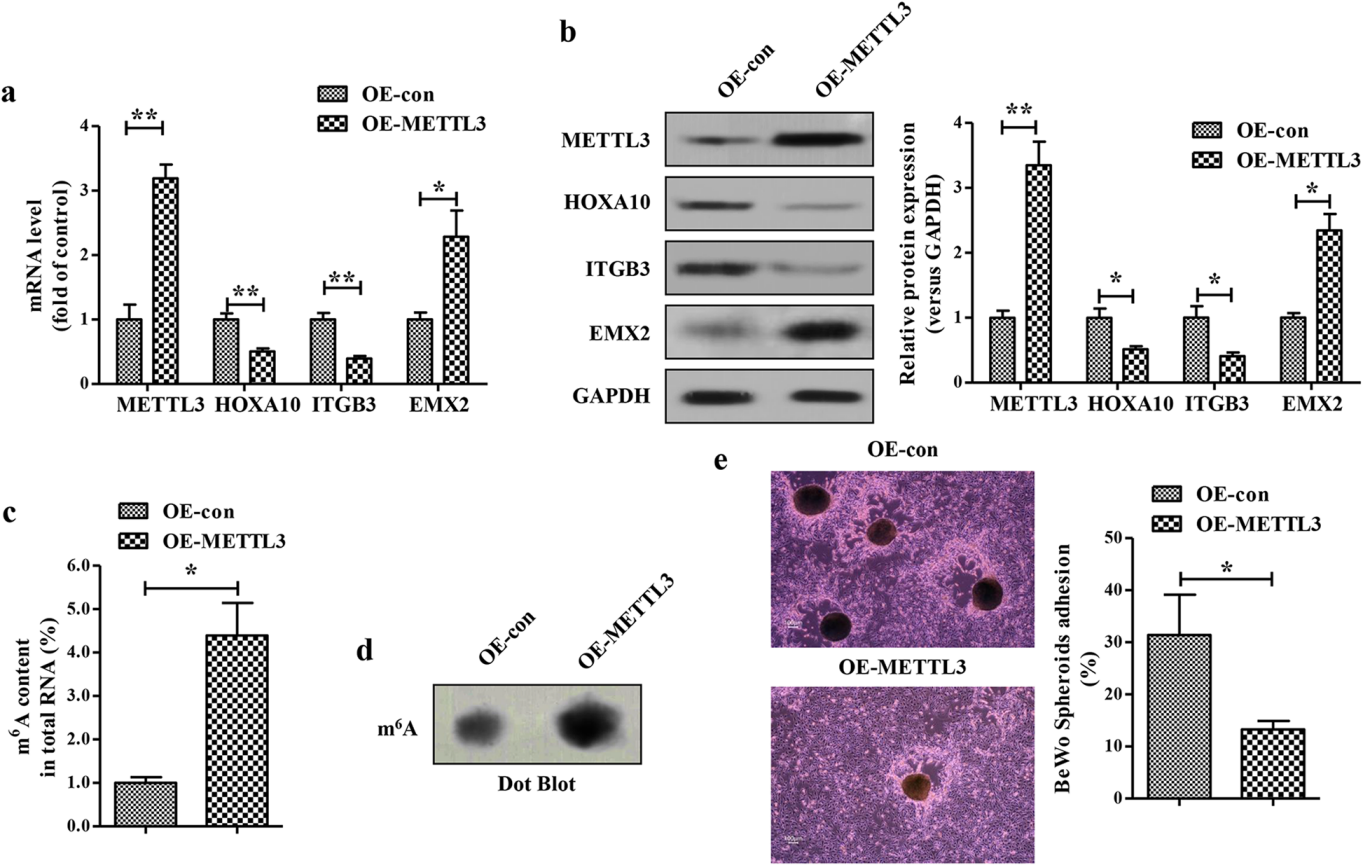
**METTL3 overexpression impairs embryo attachment** ***in vitro*. a-b** The expressions of METTL3, HOXA10, ITGB3 and EMX2 at the mRNA (**a**) and protein (**b**) levels in the METTL3-overexpressing Ishikawa cells were analyzed by qRT-PCR (**a**) and Western blotting(**b**), respectively. **c** The levels of m^6^A RNA methylation in the METTL3-overexpressing Ishikawa cells were evaluated by m^6^A RNA Methylation Assay Kit. **d** The levels of m^6^A in the METTL3-overexpressing Ishikawa cells were evaluated by dot blotting assay. **e** A vitro model of a confluent monolayer of Ishikawa cells co-cultured with BeWo spheroids was used to evaluate the embryo attachment. Bar 100 μm. The data are the average of three independent experiments (n=3). *P<0.05, **P<0.01 versus the indicated group

To further investigate the effects of METTL3-mediated m^6^A modification on embryo attachment, we established METTL3 overexpression cell models in Ishikawa cells by lentivirus. The efficiency of overexpressing METTL3 at the mRNA and protein levels were verified by qRT-PCR (Fig. 2a) and Western blotting (Fig. 2b), respectively. We found that METTL3 overexpression significantly enhanced total m^6^A levels in Ishikawa cells, as indicated in the colorimetric m^6^A quantification assay (Fig. 2c) and dot blotting assay (Fig. 2d). In an vitro model of a confluent monolayer of Ishikawa cells co-cultured with BeWo spheroids, METTL3 overexpression significantly decreased the ration of BeWo spheroids attachment (Fig. 2e). As HOXA10 is a well-characterized marker of endometrial receptivity and a critical upstream regulator of ITGB3 and EMX2, we then evaluated the expressions of HOXA10 and its downstream targets in METTL3-overexpressing Ishikawa cells. Notably, significant decreases in both HOXA10 mRNA (Fig. 2a) and protein (Fig. 2b) levels were observed in METTL3-overexpressing Ishikawa cells. Moreover, the METTL3-overexpressing Ishikawa cells exhibited a lower expression of ITGB3 and a higher expression of EMX2 compared with the control cells (Fig. 2a and b). Collectively, METTL3 may impair embryo attachment *in vitro* by inhibiting HOXA10 expression.

### METTL3 epigenetically decreases HOXA10 expression

**Fig. 3:**
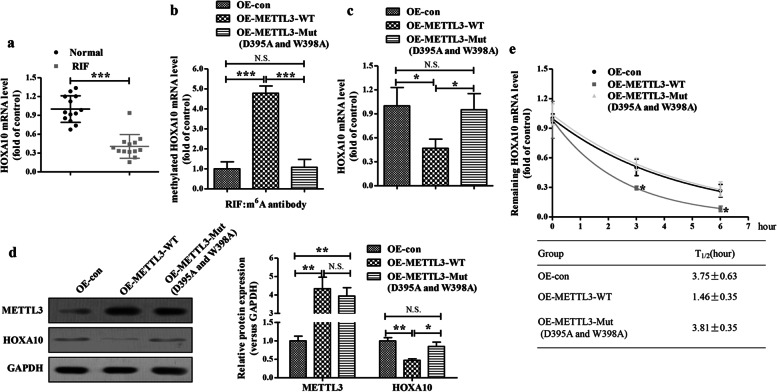
**METTL3 epigenetically decreases HOXA10 expression. a** The mRNA levels of HOXA10 in the endometrial tissues from RIF patients (n=13) and normal control women (n=13) were evaluated by qRT-PCR. **b** The methylation of HOXA10 mRNA in the wild-type/mutant (D395A and W398A) METTL3-overexpressing Ishikawa cells were analyzed by Me-RIP-qRT-PCR. **c-d** The mRNA and protein levels of HOXA10 in the wild-type/mutant (D395A and W398A) METTL3-overexpressing Ishikawa cells were analyzed by qRT-PCR (**c**) and Western blotting (**d**), respectively. **e** The curve and statistical analysis of HOXA10 mRNA decay slope in the wild-type/mutant (D395A and W398A) METTL3-overexpressing Ishikawa cells after transcriptional inhibition. *P<0.05, ***P<0.001 versus the indicated group. N.S. means *P* >0.05

We next investigated the mechanism by which METTL3 participates in the regulation of HOXA10 and its downstream targets expressions, contributing to the impairment of embryo implantation. We first assessed the mRNA levels of HOXA10 in endometrial tissues from RIF and normal subjects. The mRNA levels of HOAX10 were significantly decreased in the endometrial tissues from RIF patients (Fig. 3a). Then, we analyzed transcriptome m^6^A mapping data by an online m^6^A modification site predictor (http://www.cuilab.cn/sramp/) and found that at least 11 m^6^A residues were located across the HOXA10 sequence. Consistently, METTL3 overexpression significantly enhanced m^6^A methylation of HOXA10 mRNA in Ishikawa cells (Fig. 3b). When we generated a mutated METTL3 (D395A and W398A) construct with disordered enzymatic activity, as described previously [35], we found that the mutated METTL3 (D395A and W398A) failed to elevate the m^6^A methylation of HOXA10 mRNA in Ishikawa cells (Fig. 3b). The levels of HOXA10 mRNA and protein were both decreased by wild-type METTL3, but not mutated METTL3 (D395A and W398A) in Ishikawa cells (Fig. 3c and d). Furthermore, the decay rate of HOXA10 mRNA was accelerated rapidly by wild-type METTL3, but not mutated METTL3 (D395A and W398A), in the transcription inhibition assay (Fig. 3e). These results demonstrated that METTL3 epigenetically decreases HOXA10 expression.

### HOXA10 overexpression rescues METTL3-impaired embryo attachment *in vitro*

**Fig. 4:**
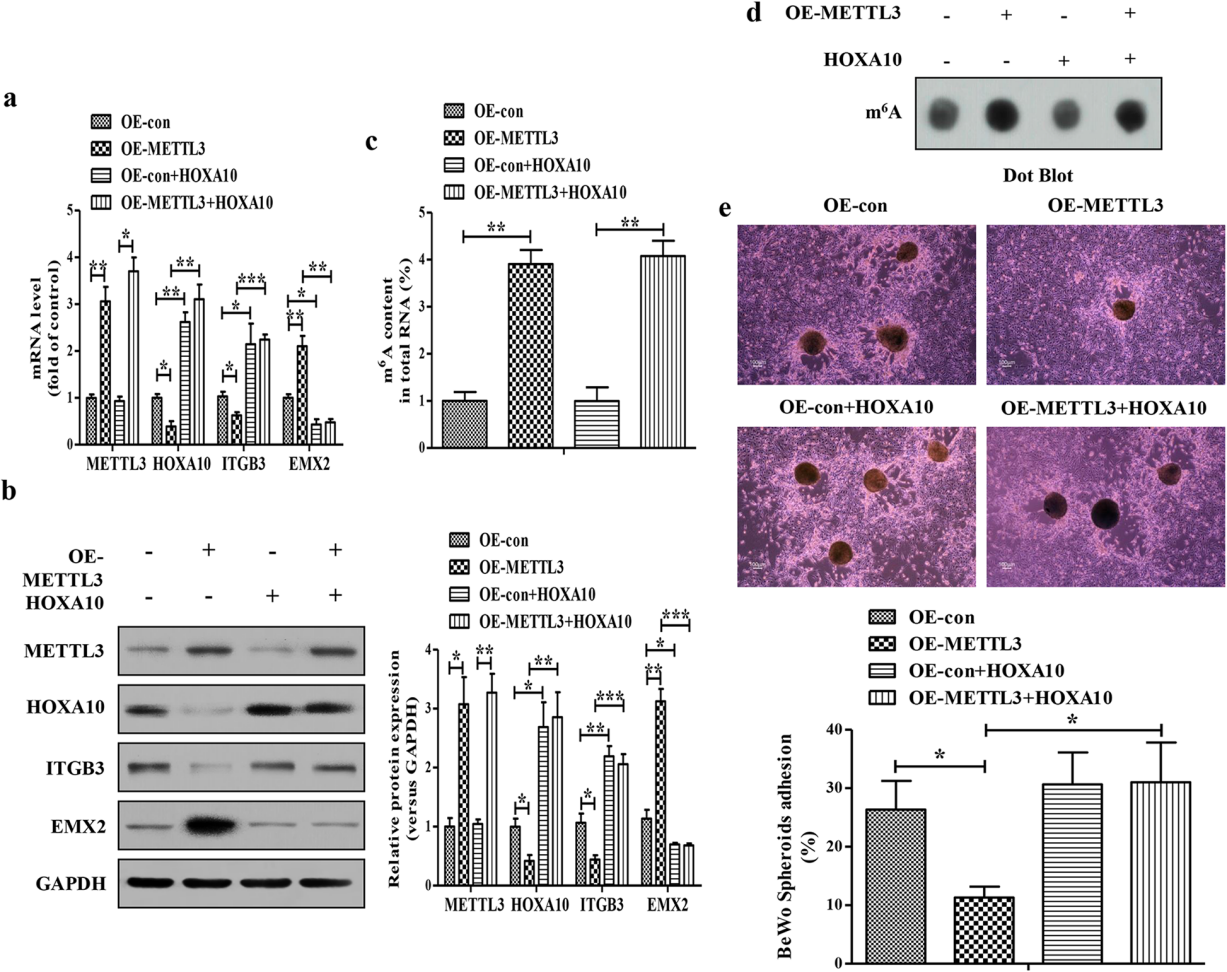
**HOXA10 overexpression rescues METTL3-impaired embryo attachment** ***in vitro*. a** The mRNA levels of METTL3, HOXA10, ITGB3 and EMX2 in the METTL3-overexpressing Ishikawa cells with or without HOXA10 overexpression were analyzed by qRT-PCR. **b** The protein levels of METTL3, HOXA10, ITGB3 and EMX2 in the METTL3-overexpressing Ishikawa cells with or without HOXA10 overexpression were analyzed by Western blotting. **c** The levels of m^6^A RNA methylation in the METTL3-overexpressing Ishikawa cells with or without HOXA10 overexpression were evaluated by the m^6^A RNA Methylation Assay Kit. **d** The levels of m^6^A in the METTL3-overexpressing Ishikawa cells with or without HOXA10 overexpression were evaluated by dot blotting assay. **e** A vitro model of a confluent monolayer of Ishikawa cells co-cultured with BeWo spheroids was used to evaluate the embryo attachment. Bar 100 μm. The data are the average of three independent experiments (n=3). *P<0.05, **P<0.01, **P<0.001 versus the indicated group

To determine whether decreased HOXA10 expression was responsible for the reduced ration of BeWo spheroids attachment upon METTL3 overexpression in Ishikawa cells, we attempted to rescue this phenotype by overexpressing HOXA10. The efficiency of METTL3 and HOXA10 overexpression were verified by qRT-PCR (Fig. 4a) and Western blotting (Fig. 4b). As above, METTL3 overexpression induced corresponding changes in the expressions of HOXA10, ITGB3 and EMX2, whereas HOXA10 overexpression dramatically enhanced the expression of ITGB3 and decreased the expression of EMX2 despite in the METTL3-overexpressing Ishikawa cells (Fig. 4a and b). We further found that overexpression of METTL3 dramatically increased total m^6^A levels with or without HOXA10 overexpression in Ishikawa cells (Fig. 4c and d). In the vitro model of a confluent monolayer of Ishikawa cells co-cultured with BeWo spheroids, HOXA10 overexpression significantly reversed the METTL3-decreased ration of BeWo spheroid attachment (Fig. 4e). Collectively, these results suggested that METTL3 impaired embryo attachment *in vitro* in a HOXA10-dependent manner.

## Discussion

With the application of high-throughput sequencing for m^6^A mapping in RNA, the understanding of its internal regulatory mechanism is being revealed. At present m^6^A has been recognized as the most prevalent internal modification in mRNAs. The m^6^A modification in mRNAs could influence mRNA stability and splicing, translation efficiency, nuclear output, and selective polyadenylation. The m^6^A modification is maintained by three different groups of RNA binding proteins, including m^6^A writers, erasers and readers. Dynamic and reversible nature of m^6^A modification makes it play a key role in cellular communications [22–32]. In the current study, we found that the levels of m^6^A-modified RNAs and the critical methyltransferase METTL3 were significantly upregulated in the endometrial tissues of RIF. METTL3 overexpression inhibited the endometrial receptivity biomarker HOXA10 expression and impaired the embryo attachment *in vitro*. These results suggested that METTL3-mediated m^6^A modification is an important determinant of embryo implantation, and that increased METTL3 expression might contribute to the pathogenesis of RIF.

Deregulation of m^6^A modification has been recently implicated in endometrial diseases [36–38]. Jiang *et al*. [36] analyzed the expressions of 20 m^6^A regulators in 34 normal, 127 eutopic, and 46 ectopic samples of endometrium tissue from different menstrual cycle phases which were merged from public microarray datasets of endometriosis, and found that most m^6^A methylation regulators in endometriosis were abnormal in the eutopic *vs.* normal endometrium, including decreased METTL3/METTL14/RBM15/FTO and increased ALKBH5. Moreover, METTL3 expression in endometriosis was reduced in the ectopic vs. eutopic endometrium while FTO expression was elevated. Functional, co-expression, correlation analyses of proliferative phase endometrial tissues from adenomyosis *vs.* controls found that decreased METTL3 expression in adenomyosis led to declining total m^6^A levels and the downstream increased insulin-like growth factor-1 (IGF1) and D-Dopachrome Tautomerase (DDT); and it revealed that IGF1 and DDT might correlate with epithelial cell proliferation and migration, both of which are involved in the pathogenesis of adenomyosis [38]. In addition, ∼70% of endometrial tumors exhibited reductions in m^6^A methylation that are due to either METTL14 mutation (R298P) or decreased METTL3 expression. Reductions in m^6^A methylation decreased the negative AKT regulator PHLPP2 expression and increased the positive AKT regulator mTORC2 expression, which contributed to increased proliferation and tumorigenicity of endometrial cancer cells through the activation of AKT pathway [37]. In the present study, increased METTL3 expression and m^6^A levels were found in the endometrial tissues from RIF. Overexpression of METTL3 increased m^6^A methylation, and impaired embryo attachment *in vitro* by inhibiting the endometrial receptivity biomarker HOXA10 expression. These studies suggest that m^6^A methylation is involved in the pathogenesis of endometrial diseases, including endometriosis, adenomyosis, endometrial cancers and RIF, all of which shares some characteristics with each other. However, different status and pattern of m^6^A methylation and its regulators may be found in the endometrial tissues from different endometrial diseases, different menstrual cycle phases, and even from different sites in the same patient.

Embryo implantation is a subtle and complicated process that requires accurate communication between high-quality embryos and receptive endometrium under the action of maternal hormones and their downstream molecules [1–3]. During ∼6 days after ovulation, ovarian estrogen and progestin cooperatively induces the morphological and physiological changes of epithelial cells in the endometrium and secretion of various cytokines. These transformations cause the uterus to be receptive to blastocyst implantation [4, 5]. The transcription factor homologue HOXA10 has emerged as an important and well-characterized biomarker of endometrial receptivity. The expression of HOXA10 in the uterus depends on the stage of menstrual cycle, which is significantly increased in the mid-secretory phase, corresponding to the implantation time and the increase of progesterone level [8, 9]. Both estrogen and progestin independently and synergically elevated the expression of HOXA10 in endometrium [8]. In turn, HOXA10 regulates endometrial acceptance and decidualization activation or compression by downstream markers specific to the window of implantation [10–12, 18–21]. Abnormal expression of HOXA10 and its downstream target genes leading to decreased endometrial receptivity are closely related to female infertility in the patients with gynecological diseases, such as endometriosis [11, 20, 21], adenomyosis [39, 40], and hydrosalpinx [41]. The importance of maternal HOXA10 expression in embryo implantation has been demonstrated by a targeted disruption of the *Hoxa10* gene in mice. Female mice with deletion of *Hoxa10* gene were infertile due to endometrial receptivity defects [42]. Small ubiquitin like-modifier 1 (SUMO1) inhibited HOXA10 protein stability and transcriptional activity via sumoylation at the evolutionarily conserved lysine 164 residue in the endometrium of women with RIF, which impairs endometrial receptivity and embryo implantation [19]. In our study, we found that wild-type METTL3, but not mutated METTL3 (D395A and W398A), decreased the expression of HOXA10 due to increases in the m^6^A methylation of HOXA10 mRNA in Ishikawa cells. Enforced expression of HOXA10 in Ishikawa cells effectively rescued the impairment of METTL3 on the embryo attachment *in vitro*. Jiang *et al*. [19] found that increased SUMO1-modified HOXA10 expression without changes of HOXA10 expression was detected in the mid-secretory endometrium of women with RIF; however, increased m^6^A content in total RNA with decreased HOXA10 expression was found in our study. The difference may be due to individual difference and limitation of sample size.

## Conclusions

In conclusion, increased m^6^A content in total RNA with high METTL3 expression was found in the mid-secretory endometrium of women with RIF compared with that of the control fertile women. METTL3 catalyzed the m^6^A methylation of HOXA10 mRNA and repressed the expression of HOXA10 leading to the impairment on embryo attachment *in vitro*. However, global RNA m^6^A methylation in the endometrium from women with RIF during the window of implantation is not restricted to METTL3/HOXA10, and further studies are required to investigate the function of m^6^A methylation in endometrial receptivity and embryo implantation.

## Data Availability

The datasets used and/or analysed during the current study are available from the corresponding author on reasonable request.
